# Correlates of stocking naloxone: a cross-sectional survey of community pharmacists

**DOI:** 10.1007/s11096-024-01773-3

**Published:** 2024-07-23

**Authors:** Rose Laing, Ting Xia, Elizabeth Grist, Jana Dostal, Suzanne Nielsen, Louisa Picco

**Affiliations:** Monash Addiction Research Centre, Eastern Health Clinical School, Peninsula Campus, Monash University, 47-49 Moorooduc Hwy, VIC 3199, Frankston, Melbourne, Australia

**Keywords:** Australia, Community pharmacy, Harm reduction, Naloxone, Opioids, Pharmacist

## Abstract

**Background:**

Provision of take-home naloxone (THN) and overdose education reduces opioid-related mortality. In Australia, from July 2022, all Australian community pharmacies were eligible to supply naloxone for free through the national THN Program.

**Aim:**

This study aimed to identify naloxone stocking rates and correlates of stocking naloxone across Australian pharmacies.

**Method:**

Data were collected from a representative sample of Australian pharmacists in Victoria, New South Wales, Queensland and Western Australia via an online survey. Data collected included pharmacy and pharmacist characteristics and services offered within the pharmacy, including needle and syringe programs, opioid agonist treatment (OAT) and stocking naloxone. Binary probit regression analysis was used to identify correlates of stocking naloxone after controlling for key covariates.

**Results:**

Data from 530 pharmacists were analysed. In total, 321 pharmacies (60.6%) reported stocking naloxone. Chain pharmacies and pharmacies that provided OAT had a greater probability of stocking naloxone (B = 0.307, 95%CI: [0.057, 0.556], and B = 0.543, 95%CI: [0.308, 0.777] respectively). Most (61.7%) pharmacists felt comfortable discussing overdose prevention with patients who use prescription opioids, and this comfort was associated with a higher probability of stocking naloxone (B = 0.392, 95%CI: 0.128, 0.655). Comfort discussing overdose prevention with people who use illicit opioids was lower (49.4%) and was not associated with stocking naloxone.

**Conclusion:**

There is scope to increase stocking of naloxone and comfort with overdose prevention, particularly through addressing comfort working with higher risk groups such as people who use illicit opioids.

## Impact statements

- Australia is one of the first countries globally to implement nationally funded pharmacist provision of naloxone, and experiences can have a significant impact on global naloxone provision and interventions.
- Independent pharmacies and pharmacies that do not provide opioid agonist treatment were associated with lower rates of stocking naloxone, suggesting that these pharmacies be  targeted to increase naloxone supply.
- National rates of stocking naloxone have increased to 60.6% since the implementation of the National Take Home Naloxone Program in 2022.
- Pharmacists are significantly more comfortable discussing overdose prevention with patients that take prescription opioids than patients that take illicit opioids.

## Introduction

Opioid related harm remains a significant public health concern in many high-income countries [1]. In the USA, opioids were responsible for 80,816 deaths in 2021 [2] and 3.3% of the US population reported using opioids within the same year [3]. In Canada, opioid related mortality rates increased by 600% between 2000 and 2017 [4]. While most opioid related deaths in the USA, Canada and UK are driven by illicit opioids, the harms caused by prescription opioids are still significant [5, 6]. Contrary to other countries, the majority of opioid related deaths in Australia are attributed to prescription opioids [7]. The number of unintentional opioid related deaths has nearly trebled since 2006, increasing from 338 to 856 in 2020 [8]. In 2020, 34% of opioid related deaths were attributed to heroin, 56% to other opioids (including prescription), and 10% to a combination of both prescription opioids and heroin [9].

Naloxone is an opioid antagonist that can be administered by a layperson to reverse an opioid overdose, preventing mortality [10, 11]. Delivered as first aid either as an intramuscular injection or a nasal spray, it can rapidly and temporarily reverse the effects of opioid overdose [12]. Take-home naloxone (THN) has been widely adopted and has consistently been associated with reduced mortality from unintended opioid overdose in many high-income countries such as the UK, USA and Australia [13–15]. In light of this evidence, in 2014 the World Health Organization (WHO) published guidelines on the use of naloxone to treat opioid overdose, and made strong recommendations that naloxone be available to all individuals who may be at risk of opioid overdose and to those who have a high chance of witnessing an opioid overdose [16]. Since these recommendations were published, THN programs have expanded globally and are now being trialled in low- and middle-income countries [15].

Globally, pharmacists have been recognized as potential educators and distributors of naloxone to the community [17]. In Australia, THN became available over the counter in 2016 [18], and implementation of the national THN program in 2022 enabled individuals to access naloxone free of charge at any participating community pharmacies [19]. However, there are still barriers to patients obtaining naloxone including lack of education on availability and usage, fear of stigma associated with opioid use and addiction [20, 21], pharmacists’ discomfort in discussing overdose prevention with patients and stigma associated with illicit versus prescription opioid use [22]. These barriers hinder pharmacists’ ability to deliver optimal pharmacy practice, resulting in evidence-based care not being available to all patients [23].

There are limited data on rates of stocking naloxone, and correlates of stocking naloxone have yet to be identified in Australia. Global literature suggests that several pharmacist and pharmacy specific characteristics may be associated with whether naloxone is stocked in a pharmacy, such as pharmacy location [24], whether the pharmacy is part of a chain [25], and provision of other services within the pharmacy [26]. Understanding factors that may serve as barriers or facilitators to naloxone supply is crucial to increasing naloxone access.

### Aim

To determine pharmacist and pharmacy-related correlates of stocking naloxone in four Australian states; Victoria, New South Wales (NSW), Queensland and Western Australia.

### Ethics approval

Ethical approval for the study was granted by the Monash University Human Research Ethics Committee (No. 36459) on 17/05/2023.

## Method

### Design and setting

Data for this analysis were collected via a cross-sectional online survey exploring pharmacists’ role in opioid use safety [27], among a representative sample from the four more populous states of Australia (Victoria, NSW, Queensland and Western Australia), representing 88% of the Australian population [28]. Results are reported according to the STROBE cross-sectional reporting guidelines [29].

### Sampling of pharmacies

A comprehensive list of community pharmacies in each state was obtained using two publicly available pharmacy marketing lists, Maven Marketing and Australian Marketing List. These lists were combined, and duplicates and non-community pharmacies were removed. This resulted in a total of 1981 pharmacies identified in NSW, 1237 in Queensland, 1469 in Victoria and 633 in Western Australia.

### Participants and procedures

**Fig. 1:**
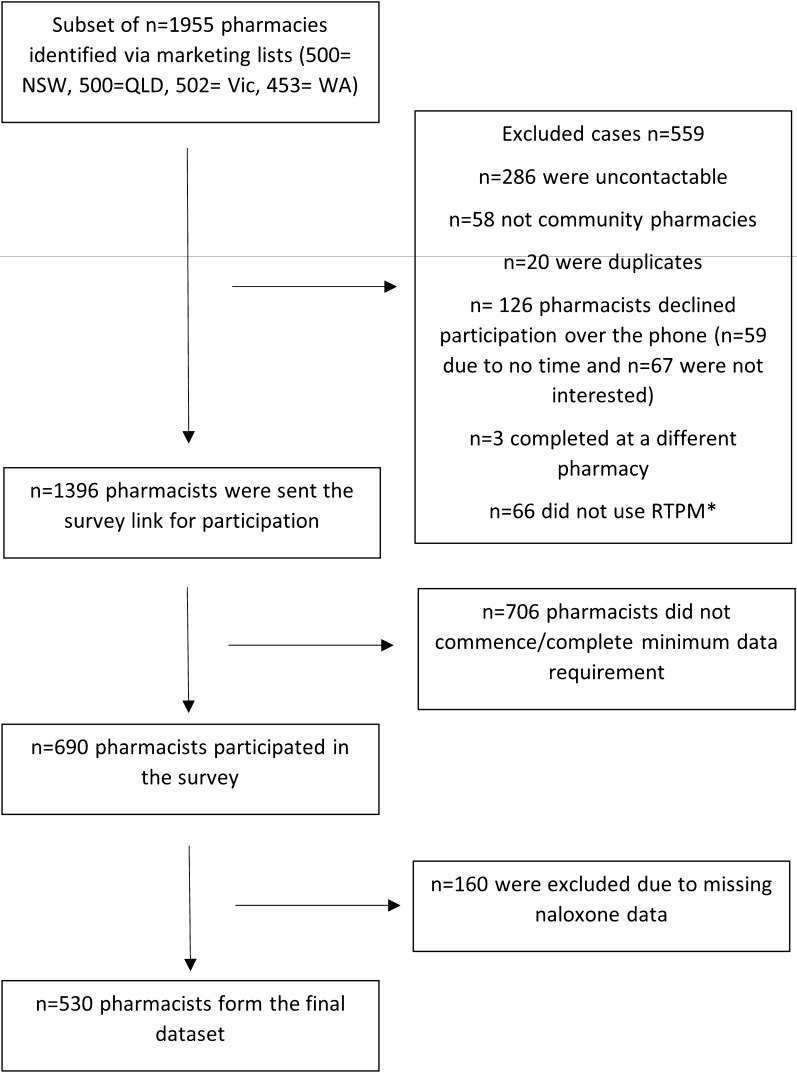
Flow diagram of participant recruitment. *Note*. * Pharmacists were required to be using their states’ real time prescription monitoring system to participate

Recruitment occurred throughout August–October 2023. Minimum sample size was calculated using ‘Raosoft”, an online sample size calculator [30]. The total population of n = 5320 identified community pharmacies was used in the calculation with a confidence level of 95% and a margin of error of 5%, with an estimated response rate of 50%, indicated from a previous study [31]. Minimum sample size was calculated at n = 359. Approximately 500 pharmacies per state were randomly selected. Equal numbers of pharmacies from each state were contacted to ensure that each states sample size was sufficient enough to make comparisons and examine correlates in naloxone stocking rates between jurisdictions. The total population of pharmacies was divided into subsets by state, and each subset was randomised using the Microsoft Excel formula “ = rand()”. A total of 1955 pharmacies were approached via telephone, based on an estimated response rate to provide the required sample size (see Fig. 1) [32]. Once contacted, the pharmacist in charge was invited to take part in the survey, with only one pharmacist per pharmacy eligible to participate to remove issues of clustering. At least three attempts were made, on different days and times, to contact the pharmacist in charge at each pharmacy. If they agreed to participate, a link to access the survey was sent to the provided email address.

The online survey was administered via Monash University Qualtrics platform. From the date of the initial email, reminder emails were sent at the one- and two-week marks to prompt participation. The participating pharmacist was required to read the associated study information sheet and provide online consent before they commenced the survey.

### Measures

The current study examined a range of potential correlates of stocking naloxone, which were selected based on existing literature [24–26, 33, 34] including 2 earlier studies with similar populations in 2016 [34] and 2020 [31], which also utilised questions from prior research [34]. The 2020 survey was cognitively tested with pharmacists, a process designed to improve the quality of survey questions through testing comprehension, retrieval, judgement, and response processes [35], with minor changes being made that are reflected in the current survey. As most covariates were recorded as binary responses, other covariates were recoded and collapsed into binary categories for consistent analysis.

### Pharmacist characteristics

*Gender:* Male or female. Originally recorded as male, female or non-binary. Non-binary was excluded from analysis due to small response rate (n = 3).

*Years of experience as a pharmacist:* < 15 years or 15 years or more. Originally recorded as a continuous variable where participants entered years of experience manually. These were divided into binary variables using mean number of years’ experience as an indicator of centrality (mean = 14.88).

### Pharmacy characteristics

*Pharmacy state:* Pharmacists in NSW, Victoria, Queensland or Western Australia were included.

*Geographic Location:* Capital city/urban and rural/remote. Originally recorded as capital city, urban, rural or remote and these were collapsed to represent urban vs rural areas.

*Pharmacy type:* Chain/banner (pharmacies that operate under the same marketing banner [36]) or independent/other. Originally recorded as a single independent pharmacy, small chain or banner group (2–9 branches), large chain or banner group (≥ 10 branches), or other. To make findings comparable to existing literature [24, 37], independent pharmacies and the ‘other’ category were combined into one variable, while small and large chain or banner pharmacies were combined into a second variable.

### Frequency of opioid dispensing

*How often opioid prescriptions were dispensed:* Less than once a day, or once or more per day. Originally recorded as once per week, multiple times per week, around once per day, several times and day, and more than 10 times a day. These were collapsed into binary variables based on mean and mode response (mean = 2.79, mode = 3.00).

### Provision of pharmacy services

*Whether the pharmacy offered or supplied the following services*: naloxone, needle and syringe program and opioid agonist treatment (OAT) (methadone and buprenorphine for opioid use disorder), which were coded as binary variables (‘yes’ if provided, ‘no’ if not provided).

### Pharmacists’ comfort

Pharmacists' comfort in discussing overdose prevention with patients who use prescription or illicit opioids: Comfortable or Not Comfortable. Originally collected on a 4-point Likert scale [38] with options ‘very comfortable’, ‘comfortable’, ‘uncomfortable’ and very uncomfortable’ collapsed to binary variables.

### Statistical analysis

Descriptive statistics were used to explore and describe sample characteristics. A multivariate probit regression analysis with an adjusted coefficient was used to explore the correlates of stocking naloxone. The final model included 10 variables that included both pharmacist and pharmacy characteristics. A McNemar test was used to test the difference in pharmacists’ comfort discussing overdose prevention with patients who take prescription versus illicit opioids. All statistical tests used a p value of 0.05 to determine significance. All analysis was completed using SPSS V28.

## Results

In total, 1396 pharmacists expressed interest in participating in the study, of which 690 pharmacists commenced the survey, with 530 completing the questions relating to naloxone and were included in the current analyses. The pharmacies represented in this analysis represent a randomised sample of 9% of total community pharmacies around Australia supporting the generalizability of results. Sample characteristics are reported in Table 1. A similar proportion of male and female pharmacists were represented (47.8% female), and 40.2% of pharmacists had over 15 years of experience. Most pharmacies were located in capital cities or urban areas (71.5%), were part of a banner or chain (66.8%) and received scripts for opioids more than once per day (91.5%). Naloxone was stocked in 60.6% of pharmacies.

**Table 1:** Pharmacy and pharmacist characteristics by naloxone availability and correlates of stocking naloxone

Sample and pharmacy characteristics		Overall (*n*)^b^ *n* = 530	Stock Naloxone (*n*, %) *n* = 321 (60.6%)	Do not Stock Naloxone (Reference) (*n*, %) *n* = 209 (39.4%)	*B*	*P*	95% CI
*Pharmacist in charge characteristics*							
Gender^a^	Male (52.2%)	275	176 (64.0%)	99 (36.0%)			Ref
	Female (47.8%)	252	144 (57.1%)	108 (42.9%)	− 0.081	0.487	− 0.311, 0.148
Years of Experience as a pharmacist	< 15 years (59.8%)	317	199 (62.8%)	118 (37.2%)			Ref
	≥ 15 years (40.2%)	213	122 (57.3%)	91 (42.7%)	− 0.079	0.505	− 0.312, 0.154
*Pharmacy characteristics*							
State	New South Wales (22.3%)	118	63 (53.4%)	55 (46.6%)			Ref
	Victoria (27.7%)	147	92 (62.6%)	55 (37.4%)	0.254	0.135	− 0.079, 0.587
	Queensland (30.2%)	160	103 (64.4%)	57 (35.6%)	0.160	0.329	− 0.162, 0.483
	Western Australia (19.8%)	105	63 (60.0%)	42 (40.0%)	0.136	0.453	− 0.219, 0.490
Pharmacy Location	Capital City/Urban (71.5%)	379	231 (60.9%)	148 (39.1%)			Ref
	Rural/Remote (28.5%)	151	90 (59.6%)	61 (40.4%)	− 0.011	0.934	− 0.264, 0.242
Pharmacy Type	Single/Independent (33.2%)	176	94 (53.4%)	82 (46.6%)			Ref
	Banner/chain/other (66.8%)	354	227 (64.1%)	127 (35.9%)	***0.307***	0.016^*^	***0.057, 0.556***
Number of Opioid Prescriptions per day in last week	< Once a day (8.5%)	45	24 (53.3%)	21 (46.7%)			Ref
	> Once a day (91.5%)	485	297 (61.2%)	188 (38.8%)	0.113	0.589	− 0.298, .524
Provides Opioid Agonist Treatment (OAT)	No (52.8%)	280	141 (50.4%)	139 (49.6%)			Ref
	Yes (47.2%)	250	180 (72.0%)	70 (28.0%)	***0.543***	< 0.001^**^	***0.308, 0.777***
Needle and Syringe Program	No (43.6%)	231	127 (55.0%)	104 (45.0%)			Ref
	Yes (56.4%)	299	194 (64.9%)	105 (35.1%)	0.051	0.685	− 0.195, 0.296
*Pharmacists’ comfort*							
Comfort discussing overdose prevention and naloxone with patients who are prescribed opioids	Uncomfortable (38.3%)	203	99 (48.8%)	104 (51.2%)			Ref
	Comfortable (61.7%)	327	222 (67.9%)	105 (32.1%)	***0.392***	0.004^*^	***0.128, 0.655***
Comfort discussing overdose prevention and naloxone with patients who use illicit opioids	Uncomfortable (50.6%)	268	140 (52.2%)	128 (47.8%)			Ref
	Comfortable (49.4%)	262	181 (69.1%)	81 (30.9%)	0.206	0.123	− 0.056, 0.468

*B* represents standardised coefficient. *P* represents *P* value. ^a^Participants that identified as ‘other’ too small to enable analysis, total sample size n = 527. ^b^All values represent 100%. *p < 0.05, **p < 0.001. Ref=reference group

The bolditalics within the table represent covariates significant values from statistical analysis

Chain or banner group pharmacies had a 30.7% greater probability of stocking naloxone, compared to independent pharmacies (*p* = 0.016, 95%CI [0.057, 0.556]), while pharmacies that provide OAT had a 54.3% greater probability of stocking naloxone after controlling for other covariates (*p* < 0.001, 95%CI [0.308, 0.777]) (Table 1). Pharmacists who were comfortable discussing overdose prevention with patients who use prescription opioids had a 39.2% greater probability of stocking naloxone, compared with pharmacists who indicated they were uncomfortable (*p* = 0.004, 95%CI [0.128, 0.655]). In contrast, comfort discussing naloxone with people who use illicit opioids was not associated with stocking naloxone.

A significantly greater proportion (61.7%) of pharmacists were comfortable discussing overdose prevention with patients who take prescription opioids versus those who take illicit opioids (49.4%) (X^2^  = 29.468, *p* < 0.001) *(*Table 2*).* Of those pharmacists who felt comfortable discussing overdose prevention with patients who take prescription opioids, 31.2% (n = 102) did not feel comfortable discussing overdose prevention with patients who take illicit opioids.

**Table 2:** Comparison of pharmacist comfort levels discussing overdose prevention with patients who take illicit opioids and those who take prescription opioids

		Comfort discussing overdose prevention with patients taking illicit opioids		Total
Comfortable	Uncomfortable			
Comfort discussing overdose prevention with patients taking prescription opioids	Comfortable	225	102	327 (61.7%)
	Uncomfortable	37	166	203 (38.3%)
	Total	262 (49.4%)	268 (50.6%)	530 (100%)
	X^2^			***29.468***
	*p* Value			***P* ≤ *0.001***

The bolditalics within the table represent covariates significant values from statistical analysis

## Discussion

### Statement of key findings

This study, among a representative sample of pharmacies in four Australian states, examined stocking naloxone and its correlates. Most (60.6%) pharmacies stocked naloxone, though as almost 40% of pharmacies did not yet stock it, there is still scope to further increase access to naloxone among these pharmacies. Results revealed that pharmacies within a banner group or chain and those that offer OAT had a greater probability of stocking naloxone. Similarly, pharmacists who were comfortable discussing overdose prevention with patients who use prescription opioids had a greater probability of stocking naloxone. Lower levels of comfort were reported with discussing naloxone and overdose education with people who use illicit opioids compared to people who were prescribed opioids.

### Strengths and weaknesses

This is the first Australian study to explore stocking naloxone in community pharmacies since the introduction of the national THN program. Strengths include the use of a large, randomly selected representative sample of pharmacies from four Australian states with diverse approaches to naloxone provision.

Health care professional surveys commonly have low response rates, so several evidence-based strategies were used to maximise responses [39]. As a result of these strategies, 49.4% of pharmacists started the survey, with 38% completing the section relating to stocking naloxone. A strength is the use of cognitive testing and existing survey questions used in previous national and international studies [31, 34] which enable comparisons to the global literature. We note that a limitation in general is a lack of already validated instruments to measure these outcomes. Collapsing multivariant responses, while assisting interpretation, may also result in a loss of data precision.

Additional limitations include a lack of information on current naloxone provision, noting that previous research found pharmacies may stock naloxone, however, were not providing or supplying it to patients [34]. There is the possibility that social desirability bias may have influenced results, as pharmacists may have reported feeling comfortable discussing overdose prevention while they were not, however the significantly lower rates of comfort when supplying naloxone to people who use illicit drugs suggest that this was not likely to have overly influenced results. Selection and attrition bias may be present from the pharmacists that declined to participate or did not complete the survey, as they may display certain characteristics that are not represented in the analysis. Due to the design of the study, a temporal relationship between pharmacists’ comfort and decisions to stock naloxone cannot be determined.

### Interpretation

In the past, stocking naloxone was not common, with a 2016 Australian study identifying that only 23% of pharmacies stocked naloxone and only 6% had dispensed it to a patient to take home. A more recent study amongst 265 pharmacists in Victoria, Australia found that 38% of pharmacies stocked naloxone, however a third of those did not supply it in the past year [31]. This study shows that rates of stocking naloxone have increased substantially in recent years, coinciding with the national THN program that covers the cost of naloxone being provided at no charge to pharmacy patients, alongside a small dispensing fee. Despite this positive change, our findings also indicate that there are still a large number of pharmacies that are not stocking naloxone, and are not comfortable with naloxone supply, with inconsistent naloxone availability having the potential to reduce community access to evidence-based care for overdose prevention.

Consistent with earlier research [31], provision of OAT was significantly associated with stocking naloxone, and pharmacists who provided OAT had a significantly greater probability of stocking naloxone. As pharmacists who provide OAT have higher exposure to patients who take both illicit and prescription opioids, it is not surprising that they are more likely to stock naloxone. Use of naloxone is recommended in most Australian state OAT guidelines, including NSW, Victoria and Western Australia [40], and promotion of naloxone through these guidelines contributes to increased pharmacist knowledge surrounding naloxone usage for prescription opioids and may be a contributing factor to the observed higher odds of stocking naloxone.

Pharmacies within a chain or banner group had a greater probability of stocking naloxone than independent pharmacies, which is consistent with findings from the USA [41]. This pattern is likely related to differences in management structures between chain and independent pharmacies. Banner and chain pharmacies have centralised management structures, and the introduction of new state and national naloxone policies may be rapidly implemented across a large number of pharmacies as a result [42]. Centralised management structures also contribute to more effective stock control mechanisms for long term storage of naloxone, which protects them against national shortages which have been a problem across Australia [43, 44].

Additional benefits of pharmacists working in a chain pharmacy include greater access to educational resources and peer coaching opportunities [45]. Increased training and internal coordination have been shown to increase levels of stocking and providing naloxone [46]. Additionally, a 2021 Australian study investigating pharmacists’ comfort in discussing overdose prevention with people who take prescription opioids revealed that pharmacists from chain and banner pharmacies had 1.5 times greater odds of feeling comfortable discussing overdose prevention than pharmacists from independent pharmacies [47]. This suggests that efforts to support independent pharmacies to provide pharmacist training and education on the benefits of naloxone may be warranted.

Pharmacists who were comfortable discussing overdose prevention with patients prescribed opioids had a greater probability of stocking naloxone. Additionally, pharmacists were significantly more comfortable discussing overdose prevention with patients that take prescription opioids versus those that take illicit opioids. This difference may imply the presence of pharmacist stigma surrounding illicit opioid use and naloxone. Naloxone stigma is often a reflection of stigma surrounding opioid use disorder and previous research has found that pharmacists reported a fear that stocking naloxone may attract the ‘wrong clientele’ [48, 49]. Education is a proven method of reducing both opioid use disorder and naloxone related stigma [50, 51]. Although it is promising that pharmacists appear to have greater comfort discussing naloxone with people receiving prescription opioids, there is an urgent need to maximise pharmacist comfort in discussing naloxone with all people at risk of experiencing or witnessing an opioid overdose.

As one of the first countries to implement a fully funded national THN program via community pharmacies, these findings may inform expansion of pharmacy naloxone supply in other global settings. Findings also highlight the need to address stigma and increase pharmacists’ comfort in discussing naloxone with a wide range of populations, while also demonstrating the importance of funded programs to increase naloxone availability.

### Further research

While the rate of pharmacies stocking naloxone has increased across Australia, further work to understand naloxone provision and later usage is warranted. Future work could assess if current pharmacist naloxone training is sufficient to support pharmacists to feel confident supplying naloxone to different populations and identify how perceived barriers in providing naloxone can most effectively be addressed [48, 52].

## Conclusion

Despite naloxone being free under the national THN program, almost 40% of pharmacies do not stock naloxone. Additional efforts are needed to increase naloxone stocking in independent pharmacies and among those that do not offer OAT, to maximise naloxone access within the community. Pharmacists were significantly less likely to feel comfortable discussing overdose prevention with patients that take illicit opioids versus prescription opioids, and additional education may be required to increase pharmacists’ comfort in discussing naloxone with all populations.
